# Internal fixation of femoral shaft fractures in children by intramedullary Kirschner wires (a prospective study): its significance for developing countries

**DOI:** 10.1186/1471-2482-5-6

**Published:** 2005-03-29

**Authors:** Shashank D Chitgopkar

**Affiliations:** 1 Department of Orthopaedics, King Khalid hospital, Najran, Saudi Arabia

## Abstract

**Background:**

To evaluate internal fixation by intramedullary Kirschner wires as a surgical technique in the treatment of femoral shaft fractures in children by a prospective study.

**Methods:**

17 femoral shaft fractures at various levels in 16 children aged 2–15 years were treated by closed intramedullary Kirschner wiring under image intensifier control between May 2000 and October 2003. No external splint was used.

**Results:**

Fracture union was achieved in 6–14 weeks. Non-weight bearing crutch walking was started 2–3 days after surgery. Full weight bearing started 6–14 weeks. Average operative time was 40 min (range 20–72 min). Wires were removed after 8–22 weeks. There were no infections, no limb length disparity. One child had pin track ulceration. A big child of 14 years had angulation of the fracture.

**Conclusion:**

Intramedullary nailing of femoral shaft fractures in children by stainless steel Kirschner wires is an effective method, which compares well with other studies. It is a simple procedure, which can be easily reproduced. Blood loss is minimal, and the operative time short. There is no need pre-bend the wires in a C or S curve. Stainless steel Kirschner wires are cheap, universally available, and can be manufactured locally. The cost of Image intensifiers is affordable in most of the cities of the developing countries. The hospital does not have to maintain a costly inventory. Provides early mobility, return to home and, school. Gives a predictable clinical pathway and reduces occupancy of hospital beds.

The technique was successfully applied for internal fixation of other diaphyseal fractures in children and some selected diaphyseal fractures in adults. Based on my experience and a review of the literature, I recommend this technique as a modality for treatment of femoral shaft fractures in children aged 2 to 14 years.

## Background

This technique has been used successfully at King Khalid hospital, Najran, Saudi Arabia since 1995.

This prospective study was prompted by a review of earlier reports by Shakeel [1], Salem Al Zahrani [8], and Ligier and Metazieau [6].

Fracture treatment in children relies on rapid healing and spontaneous correction of angulated fractures; therefore most of the diaphyseal fractures can be treated by plaster alone. Operative treatment of children's fractures is often looked at critically [2].

Conventional treatment of femoral shaft fractures in children is by traction followed by a hip spica or a Thomas' splint. Conservative treatment of femoral shaft fractures gives good results in children under 5 years of age. But above that age, all such fractures cannot be treated by conservative methods. There is a possibility of loss of reduction and malunion. Plaster immobilisation has its own complications like pressure sores, nerve palsies, soiling of the skin and the plaster, breakage of the plaster, joint stiffness. The child is immobilised and needs an attendant for personal care.

Reeves et al [22] reported that the cost of non-operative treatment is 40 % higher than operative treatment.

In the last few decades, the trend worldwide has been towards some form of fixation for children's fractures, especially the femoral shaft, and the indications for operative management have been widened.

Rush [3] used the intramedullary rods of his design. Intramedullary nailing was made popular by Ender and Simon-Weidner in Europe [4], and by Pankovitch in the United States [5].

Beaty, Austin and Canale [12] studied the preliminary results and complications of interlocking intramedullary nailing of femoral shaft fractures in adolescents.

Gonzalez and Herranz[13] recommend the avoidance of rigid intramedullary nails introduced through the piriformis fossa in children less than 13 years of age. Antegrade intramedullary nailing through the piriformis fossa may cause coxa valga, epiphyseodesis of greater trochanter, thinning of the femoral neck because of damage to the growth plate[13].

Saxer[14] advises the introduction of intramedullary Kuntscher nail through the sub-trochanteric zone or the use of plate and screw.

External fixation has been advocated by Aronson and Tursky[9]. They reported angular deformity and shortening of more than 13 mm in proximal third femoral shaft fractures treated by conservative means. External fixation has its own complications; pin track infection and refracture[9,18,19]. Also, the child has to accommodate an external device.

Compression plating was used by Van Neikerk [10], Ward [11] and Hansen [17]. The disadvantages are the risk of infection, large soft tissue dissection, delayed union, limb length disparity, another large exposure to remove the implants [10,11]. The other disadvantages are periosteal stripping, evacuation of fracture haematoma and blood loss. Tarek Mirdad [21] reported blood loss requiring blood transfusion in 41 % of children treated by compression plating. Also, a period of 3–4 weeks of protected weight bearing is recommended after removal of plate and screws.

Ligier and Metaizeau have successfully treated 123 femoral shaft fractures in children by Elastic Stable Intramedullary Nailing (ESIN) [6].

Pradeep Kumar [7], Shakeel [1] and Zahrani [8] recommend the efficacy of Kirschner wires for flexible intramedullary nailing of femoral shaft fractures in children. Shakeel reports reduced psychological trauma on the child and the parents [1].

## Methods

Between May 2000 and October 2003, 17 femoral shaft fractures at various levels in 16 children aged 2–15 years were treated by internal fixation with intramedullary Kirschner wires at King Khalid hospital, Najran, Ministry of Health, Saudi Arabia.

### Inclusion criteria

a. Displaced fractures, with or without comminution.

b. Multiple fractures.

c. Fractures found to be unstable on closed reduction.

d. Fractures which displaced in traction.

e. Fractures in patients with polytrauma and patients under intensive care to facilitate nursing.

f. Irritable patients with brain injury.

g. Associated vascular injury needing repair.

### Exclusion criteria

Undisplaced fractures and fractures in a good position were treated by traction and hip spica.

### The goal

To provide rapid healing of the fracture in a correct position, ease of nursing care, early mobilisation, and return to home and school.

### Operative technique

Under general anaesthesia, the patient was placed supine on an orthopaedic table with the feet strapped to the footplates and longitudinal traction applied ensuring correct linear and rotational alignment clinically and radiologically using an image intensifier. 3 small children were operated upon a radiolucent table, traction being applied by an assistant.

**Figure 1:**
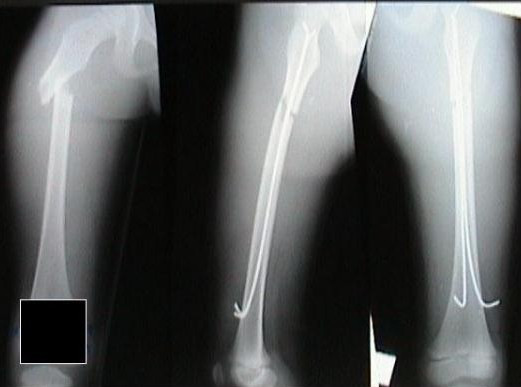
Proximal femoral shaft fracture.

2 stainless steel Kirschner wires of 30–45 cm length and 2.5–3.5 mm diameter (depending on the size of the medullary canal and the child) were prepared by bending them at an approximate angle of 45°, 2 cm from the tip and cutting off the sharp points to prevent inadvertent penetration of the cortex. The wires were not pre-bent in a 'C' or 'S' curve. The wires were loaded onto a 'T' handled introducer with a Jacob's chuck. Two small skin incisions were made proximal to the superior pole of the patella, one laterally and the other antero-medially. Entry portals were made into the distal femoral metaphysis proximal to the growth plate of the distal femur with a sharp bone awl laterally and antero-medially. The wires were introduced retrograde by hand or gentle hammering. The lateral wire was introduced first. The tips of the wire are placed just distal to the growth plates of the greater and lesser trochanters, the bends pointing towards the side of the entry portal [Figure 1]. Angular and rotational malalignment spontaneously corrects on passage of the wires across the fracture.

**Figure 2:**
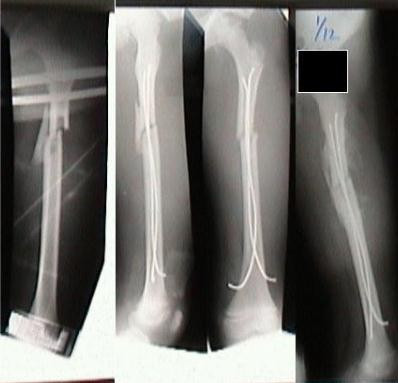
Comminuted proximal femoral shaft fracture.

Image intensifier screening in two planes perpendicular to each other confirmed proper placements of the wires. The tail portions of the wires were bent towards the fracture and cut 1 cm away from the entry portal in the cortex [Figure 2]. The skin wounds were sutured and dressed. Fracture stability, correct linear and rotational alignment was assessed on table.

All patients had received a pre-operative bolus of intravenous antibiotic.

Antegrade medullary Kirschner wiring was carried out in one small child with a single intramedullary Kirschner wire. The entry portal was on the lateral cortex distal to the growth plate of the greater trochanter.

### Post-operative rehabilitation

No external splint was used except for 2 patients who had ipsilateral tibia and fibula fractures, which were being treated by plaster immobilisation in an above knee plaster cast.

The children were started on non-weight bearing crutch walking and knee physiotherapy 2–3 days after the surgery and were ready for discharge 3–4 days after surgery. The children with polytrauma stayed on for a longer period in the hospital because of associated injuries. Sutures were removed on the 10^th ^day after surgery.

The children were encouraged to attend school three weeks after surgery avoiding sports and physical training. The school authorities consented to this and allowed an attendant for the children.

Full weight bearing was allowed after clinico-radiological fracture union. Union was defined clinically by the absence of bony tenderness and abnormal mobility at the fracture site, and no pain at the fracture site on weight bearing. Radiological fracture union was defined by the presence of callus bridging the fracture and partial obliteration of the fracture line in 2 views perpendicular to each other.

The children were assessed for malunion both linear and rotational, and limb length disparity.

The wires were removed under general anaesthesia with the help of pliers, which can be locked onto the wire and hammered on.

## Results

The children were aged 2 years to 15 years. There were 7 left sided and 8 right-sided fractures, and 1 bilateral. 14 children had met with a road traffic accident and 2 had a fall from a height. All were closed fractures [Table 2].

**Table 2:** Details of patients

Patient	Age	Sex	Side	Open/closed	Level of #	Operative time In min	Open/closed Reduction	Stay fixation In days	Stay removal In days	Weight bearing In weeks	Time # union In weeks	Time removal In weeks	Discharge In weeks
1	6	M	L	C	U3-M3 jn	47	C	5	2	6	6	13	17
2	7	M	L	C	U3	35	C	19	3	6	14	20	24
3	12	F	Bil.	C	M3	75	C	13	3	6	10	20	30
4	2	M	L	C	U3	43	C	13	Lost	To	Follow	up	-
5	14	M	R	C	M3	72	O	13	4	12	12	20	20
6	12	M	R	C	U3	36	C	06	3	14	14	22	22
7	8	M	R	C	U3-M3 jn	50	C	11	2	8	8	16	26
8	10	M	L	C	M3 com	46	C	11	3	6	6	16	24
9	10	M	L	C	M3	35	C	12	2	8	8	20	20
10	3	M	L	C	U3 com	25	C	12	Lost	To	Follow	up	-
11	11	M	R	C	M3-L3 jn	40	O	61	2	8	8	20	20
12	4	M	R	C	M3	20	C	8	6	6	6	14	16
13	14	M	R	C	M3	32	C	5	IL nail	-	-	-	-
14	3 1/2	M	L	C	L3	46	O	5	2	4	6	8	10
15	4 1/2	F	R	C	M3-L3 jn	45	C	6	2	6	6	10	12
16	15	M	R	C	M3	26	C	7	2	8	10	12	14

**Figure 3:**
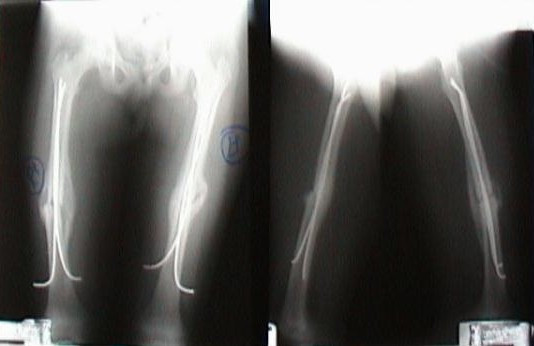
Bilateral femoral shaft fracture.

There were 6 subtrochanteric, 8 midshaft and 3 distal fractures. One child had bilateral femoral shaft fractures [Figure 3]. 3 fractures were communited. 12 children had associated injuries and 10 children had polytrauma [Table 1].

**Table 1:** Associated injuries

*Associated injuries*	*Patients*
Eye abrasion	1
Ipsilateral tibia and fibula fractures	2
Humerus fracture	1
Blunt abdomen injury	5
Blunt chest injury	1
Brain injury	7
Clavicle fracture	1
Facial injury	1
Ipsilateral femoral artery tear	1

The average operative time was 40 min (range 20–72 minutes). Open reduction had to be carried out in 3 fractures, one for an associated femoral artery tear, which needed repair. The other two were for muscle interposition. A percutaneous bone lever was introduced into the fracture site to reduce another fracture. Open reduction needed a minimum exposure with minimum blood loss, minimum handling of the periosteum and, no increase in the morbidity or alteration in the post-operative rehabilitation.

Hospital stay for fracture fixation was between 5–13 days, children with polytrauma stayed longer for treatment of associated injuries. Hospital stay for wire removal was between 2–5 days. Clinico-radiological fracture union was achieved between 6–14 weeks at which time the children started full weight bearing. The wires were removed between 8–22 weeks [Table 2].

### Complications

Pin track ulceration in 1 child. Pins were trimmed under local anaesthesia and the wounds healed in a week's time.

Exuberant callus formed in 2 children with brain injury, but this did not affect the final outcome.

The fracture angulated in a big child of 14 years. The wires were removed and a closed interlocked medullary nailing was carried out. The fracture went on to union without any further complications.

No infections were encountered.

All the children had a complete range of movement of the hip and the knee joints.

No shortening. Lengthening cannot be commented on, as this was a short-term study.

Criteria for malunion [16]

More than 15° angulation in coronal plane, 20° in sagittal plane, and 10° in rotational malalignment is unacceptable. This is variable with age with as much as 45° angulation in sagittal plane being acceptable in infants due to the remodelling potential.

## Discussion

Long years of experience and study have established the place of surgical management of femoral shaft fractures in children. Of the various modalities, ESIN is proving to be the best surgical option.

The aim is to encourage the formation of bridging periosteal callus. The wires, the bone and the muscles provide the stability. The muscles act as guy ropes, so even in an irritable child or the hyperactive child, the muscle activity just complements the fixation. Muscle action also causes spontaneous correction of any angular deformities. Micromotion allowed by the elasticity of the fixation promotes external bridging callus. The periosteum is not disturbed and being a closed procedure there is no evacuation of the fracture haematoma or risk of infection. Callus formation is twice as fast as with conventional methods [6].

In this study the wires were not pre-bent to a 'C' or 'S' curve. Kiely [20] studied the construct of C, S and straight intramedullary wires in a model simulating a fractured femur of a 6 years old child. He concluded that the principle of flexible nailing does not apply to children's femoral shaft fractures. The factor may be the small diameter of the model used in the study. He also concluded that any of the described nail combinations could be used to stabilise a small diameter bone. This provides the surgeon with a greater range of options when managing fractures. The present study agrees with these findings.

In this study, retrograde wiring was used for 2 distal fractures and a single ante grade wire for another distal fracture achieving good results. Other researchers have not reported operating on a child as young as 2 years.

In this study, children were allowed to continue normal activities after removal of the wires.

This short-term study compares well with other studies on treatment of femoral shaft fractures in children by both conservative and operative methods. It agrees with other studies on elastic nailing with titanium nails and stainless steel Kirschner wires.

Staheli [15] defined the ideal treatment of femoral shaft fractures in children as one that controls alignment and length, does not compress or elevate the extremity excessively, is comfortable for the child and convenient for the family, and causes the least negative psychological impact possible. This technique comes close to achieving this ideal.

## Conclusion

Intramedullary nailing of femoral shaft fractures in children by stainless steel Kirschner wires is an effective method, which compares well with other studies. It is a simple procedure, which can be easily reproduced. Blood loss is minimal, and the operative time short. There is no need pre-bend the wires in a C or S curve. Stainless steel Kirschner wires are cheap, universally available, and can be manufactured locally. The cost of Image intensifiers is affordable in most of the cities of the developing countries. The hospital does not have to maintain a costly inventory. Provides early mobility, return to home and, school. Gives a predictable clinical pathway and reduces occupancy of hospital beds.

The technique was successfully applied for internal fixation of other diaphyseal fractures in children and some selected diaphyseal fractures in adults. Based on my experience and a review of the literature, I recommend this technique as a modality for treatment of femoral shaft fractures in children aged 2 to 14 years.

## Competing interests

The author(s) declare that they have no competing interests.

## Pre-publication history

The pre-publication history for this paper can be accessed here:


